# Artificial intelligence in the diagnosis of deep vein thrombosis: A scoping review

**DOI:** 10.1371/journal.pone.0351558

**Published:** 2026-06-22

**Authors:** Teresa Chen, Ranita Hisham Shunmugam, Samantha Ying Ying Tan, Sheron Sir Loon Goh, Yet Yen Yan, Kwan Hoong Ng

**Affiliations:** 1 Department of Diagnostic Radiology, National University Hospital, National University Health System (NUHS), SingaporeSingapore; 2 Department of Library and Information Science, Faculty of Arts and Social Sciences, Universiti Malaya, Kuala Lumpur, Malaysia; 3 Department of Diagnostic Radiology, Singapore Health Services (SingHealth), Singapore, Singapore; 4 Department of Clinical Pharmacy and Pharmacy Practice, Faculty of Pharmacy, Universiti Malaya, Kuala Lumpur, Malaysia; 5 Department of Diagnostic Radiology, Mount Elizabeth Hospital, SingaporeSingapore; 6 Department of Biomedical Imaging, Faculty of Medicine, Universiti Malaya, Kuala Lumpur, Malaysia; 7 Department of Medical Imaging and Radiological Sciences, Kaohsiung Medical University, Kaohsiung, Taiwan; Chinese Center for Disease Control and Prevention, CHINA

## Abstract

Deep vein thrombosis (DVT) is the formation of thrombi in the deep venous system, most often in the lower extremities. Although usually not life-threatening, DVT requires timely diagnosis to prevent complications such as pulmonary embolism and post-thrombotic syndrome. The growing demand for image interpretation has generated interest in applying artificial intelligence (AI) to automated DVT detection. This scoping review analyzes the performance of artificial intelligence in diagnosing DVT using computed tomography (CT), magnetic resonance imaging (MRI), and ultrasound (US). We conducted a search across seven databases from inception to May 2025 using terms related to deep vein thrombosis, artificial intelligence, and machine learning. Eligible studies were limited to those evaluating DVT diagnosis using CT, MRI, or ultrasound. Two independent reviewers selected eligible studies, and quality was assessed using the Quality Assessment of Diagnostic Accuracy Studies (QUADAS-2). Eleven studies published between 2021 and 2025 met the inclusion criteria. Some of the AI algorithms included RetinaNet, Deep R-Belief Neural Networks, and Sooty Tern Optimization. US-based models were the most studied algorithms, with sensitivities and specificities ranging from 68 to 100% and 70–100%, respectively. The MRI-based model achieved sensitivities, specificities, and accuracies of 95% to 97%. One CT-based model demonstrated a sensitivity of 83%. Studies evaluated across multiple imaging datasets showed high sensitivities, specificities, and precision of 96% or higher. Future research should prioritize multicenter validation and integration of clinical factors. In addition, explainable frameworks capable of integrating multiple imaging datasets must be developed with attention to workflow efficiency and cost-effectiveness to support clinical translation. The results indicate that AI is best situated as a supplementary tool rather than a replacement for expert interpretation in DVT diagnosis.

## Introduction

Venous thromboembolism (VTE), comprising deep vein thrombosis (DVT) and pulmonary embolism (PE), is the third leading cause of cardiovascular death after myocardial infarction and stroke [1–3]. DVT occurs when blood clots form in the deep veins, such as those in the upper limbs, lower limbs, mesenteric, and cerebral veins [4]. However, DVT of the lower limbs constitutes the principal manifestation, with an annual incidence rate of 45–117 per 100,000 individuals in Europe and 48 per 100,000 individuals in the United States [3,5]. Although rarely fatal in isolation, lower-limb DVT is clinically significant because it can cause pulmonary embolism and post-thrombotic syndrome [3,6–10]. These conditions increase morbidity and healthcare costs, underscoring the need for early diagnosis and treatment [3,11].

Diagnostic imaging is essential for the evaluation of DVT [11]. For decades, conventional venography was the diagnostic imaging modality of choice. However, venography also poses risks of contrast media allergies, renal insufficiency, and paradoxical post-procedure DVT [11,12]. Consequently, conventional venography has lost its popularity in the field of radiology [11,12]. US is currently the first-line and most widely used imaging modality for DVT diagnosis due to its accessibility, affordability, and radiation-free nature [11]. Contemporary techniques utilize compression US, in which vein compressibility under transducer pressure indicates the presence or absence of thrombus. While vein compressibility remains the cornerstone of US diagnosis, diagnostic accuracy is strongly affected by operator expertise, patient body habitus, and local tissue conditions [11,13]. Specifically, obesity and edema can obscure venous landmarks, degrade image quality, and limit adequate compression [11,13]. Thus, diagnostic accuracy is influenced by both technical and interpretative factors. Furthermore, US is limited by its reduced sensitivity for distal DVT compared to its sensitivity for detecting proximal DVT [11,13]. US duplex demonstrates a sensitivity of 96% for diagnosing proximal DVT, 71% for distal DVT, and an overall specificity of 94% [13]. Similarly, US triplex reports a sensitivity of 94% for proximal DVT, 57% for distal DVT, and a specificity of 94% for DVT diagnosis [13].

CT and MRI may be reserved for patients with inconclusive US findings or when US has difficulty visualizing the deep veins of the abdomen and pelvis [14,15]. CT venography involves the injection of contrast into the veins, with imaging timed to match contrast enhancement in the affected region [11]. Advances in dual-energy CT (DECT) have improved venous visualization by enabling virtual monochromatic imaging (VMI) at low voltages, which enhances iodine contrast within the veins while maintaining visibility of the thrombus [16]. CT venography boasts a sensitivity of 95.9% and a specificity of 95.2% in the diagnosis of proximal DVT [17]. Nevertheless, like conventional venography, CT venography poses risks of ionizing radiation and contrast media allergies [17].

MRI may also be employed to detect DVT, as it is a non-invasive imaging modality that is not associated with radiation exposure and can achieve a sensitivity of 91.5% and a specificity of 94.8% [11,18,19]. Black-blood MRI enhances DVT diagnosis by darkening the blood signal, which improves visualization of vessel walls and intraluminal pathologies. Furthermore, MRI techniques can be categorized as indirect versus direct approaches [20]. Indirect MR venography relies on systemic gadolinium administration with the contrast arriving at the tissue of interest. Direct contrast-enhanced MR involves diluted gadolinium injected on the affected side to opacify the deep and superficial venous systems, resembling conventional venography [20]. However, limitations of this imaging modality include high costs and long imaging times [11,19].

These persistent challenges—including operator-dependent ultrasound quality, patient-related variability, the logistical constraints of CT and MR venography, and increasing clinical workload—have motivated interest in technologies capable of improving diagnostic reproducibility and workflow efficiency [21–25]. Artificial intelligence (AI) is transforming radiology by enabling automated detection, recognition, and interpretation of complex imaging features [21]. The United States Food and Drug Administration (FDA) authorization of AI and machine learning (ML) tools has expanded rapidly, with 723 radiology devices now cleared—representing 76% of all AI-enabled medical devices [26]. Many studies have reported AI models that achieved high accuracy in tasks such as cancer detection, neurodegenerative disorder detection, and stroke triage. In some cases, these models outperformed radiologists [21–25]. Beyond diagnostic accuracy, AI enhances workflow by prioritizing urgent cases, supporting preliminary reads, and assisting in report generation [21,27].

Despite AI’s widespread adoption across multiple radiological subspecialties, its application in venous thrombosis imaging presents unique technical challenges. For instance, thrombus echogenicity varies with clot age, leading to overlap with adjacent soft tissues and making automated feature extraction difficult. US images frequently contain acoustic shadowing from overlying structures such as muscle, bone, or edema, which obscures venous margins and reduces visibility of thrombus. Furthermore, lower-extremity venous US lacks standardized acquisition protocols, with wide variation in probe position, transducer pressure, limb rotation, and scanning planes between operators [28]. AI can potentially mitigate these issues by reducing operator-dependent variability, improving reproducibility, and decreasing time to diagnosis [29,30].

Lastly, existing models are limited by small sample sizes, single-center datasets, or a lack of external validation. As a result, the clinical applicability and generalizability of these systems remain uncertain. These gaps highlight the need for a comprehensive review to map current evidence, evaluate methodological quality, and identify opportunities for future development [29,30].

The included studies vary in their imaging modalities, AI algorithms, data sources, and performance metrics, limiting meaningful meta-analysis. Given the heterogeneous and emerging nature of the literature on AI in the diagnosis of DVT, a scoping review was selected over a systematic review. This review also focuses exclusively on lower-extremity DVT, which represents the most common clinical presentation of DVT.

The objective of this review is to synthesize current data on the accuracies, sensitivities, and specificities of AI algorithms for diagnosing lower-limb DVT on imaging, emphasizing existing performance gaps and potential avenues for further study.

## Materials and Methods

This review was conducted and reported in accordance with Preferred Reporting Items for Systematic reviews and Meta-Analyses extension for Scoping Reviews (PRISMA-ScR) [31]. The completed PRISMA-ScR checklist is provided in S1 Appendix. This review was registered with Open Science Framework (OSF) (osf-registrations-a5dnz-v1).

### Search strategy

A comprehensive literature search was performed to identify studies evaluating the role of artificial intelligence in detecting DVT in imaging. The search was conducted across seven major electronic databases (PubMed, CINAHL via EBSCO, Web of Science, Cochrane, Scopus, Google Scholar, and Dissertations and Theses through ProQuest) from inception to May 2025. A combination of medical subject headings and free-text terms was used for the search keywords, including ‘deep vein thrombosis,’ ‘artificial intelligence,’ and ‘machine learning.’ The full search strategy is presented in S2 Appendix. These search terms and limits were adjusted for each database to match its specifications.

### Eligibility criteria

The inclusion criteria included full-text articles that 1) focused on the use of AI algorithms for DVT diagnosis (e.g., machine learning, deep learning); 2) included adult participants over the age of 18 years; 3) involved studies using ultrasound (US), computed tomography (CT), or magnetic resonance imaging (MRI); 4) provided clear descriptions of the AI techniques used for lower limb DVT diagnosis (e.g., machine learning, deep learning); and 5) were published in all languages. The narrow inclusion criteria reflect the emerging nature of AI in DVT diagnosis, particularly for peer-reviewed studies meeting quality and methodological criteria.

The exclusion criteria included studies that 1) lacked quantitative analyses of variables of interest and 2) lacked clear descriptions of the AI algorithms used for DVT diagnosis.

### Study selection

Duplicate studies were excluded from the review. Two independent reviewers (TC and KHN) screened the titles and abstracts, assessing whether the studies met the inclusion criteria. When there was a discrepancy, discussions between the two reviewers were held to reach a consensus.

### Quality assessment and risk of bias

Scoping reviews evaluate studies with heterogeneous designs and results; therefore, thorough assessment is crucial. The Quality Assessment of Diagnostic Accuracy Studies (QUADAS-2) tool was used to evaluate the quality of the included studies [32]. The QUADAS-2 tool assesses risk for bias, applicability, and reporting quality regarding patient selection, index tests, reference standards, and flow and timing using 14 different terms [32]. The QUADAS-2 tool involves four steps: 1) review topic formulation; 2) establish guidelines for the review; 3) examine the primary study’s flow diagram; 4) assess bias and applicability [32]. The risk of bias is evaluated for each domain, and applicability issues are evaluated for the first three. Signaling questions are used to guide decisions for the risk of bias. Each item is rated as “yes,” “no,” or “unclear” [32]. The QUADAS-2 assessments of the included literature are included in S3 Appendix.

### Data extraction

TC extracted and recorded the data in a standardized extraction form. The results were verified by RHS and SSLG. Any discrepancies in extracted data were resolved through discussion among the review team. Data extracted included the publication year, study design, sample size, imaging modality, AI technique, evaluation metrics, and performance results.

## Results

### Study selection

**Fig 1:**
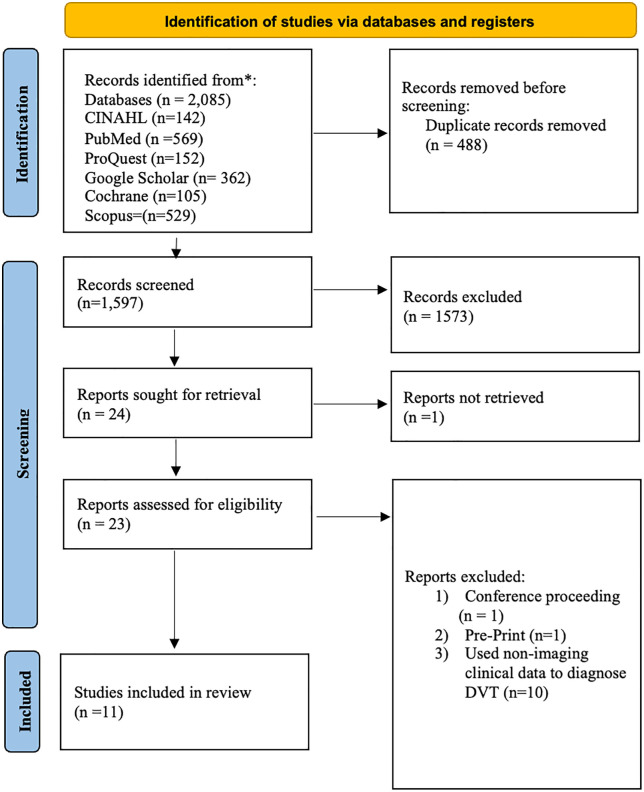
PRISMA Flow Chart for Study Selection.

The initial literature search yielded 2,085 articles. After removing duplicate studies and screening abstracts and literature, eleven studies met the inclusion and exclusion criteria (Fig. 1).

### Quality assessment

**Fig 2:**
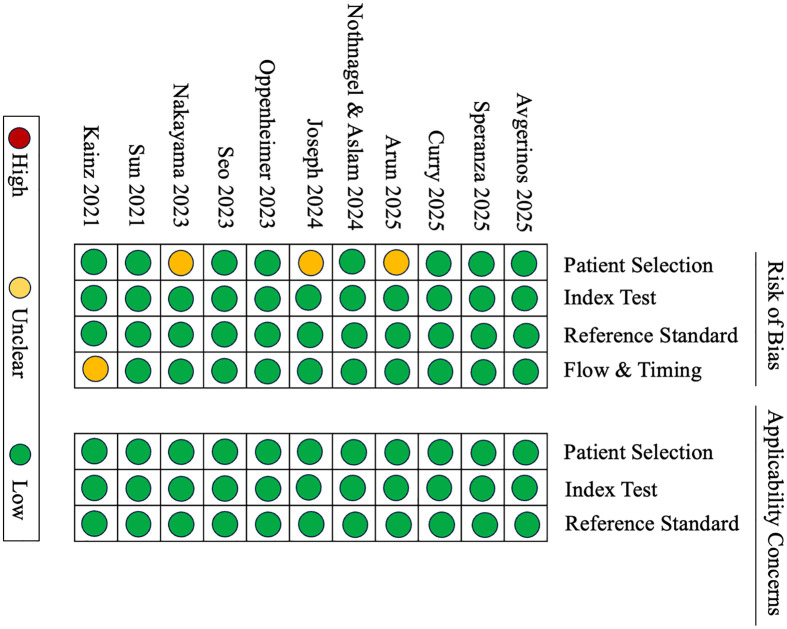
Assessment of risk of bias in studies. QUADAS-2 Tool. QUADAS-2, Quality Assessment of Diagnostic Accuracy.

The studies were analyzed using the QUADAS-2 tool (Fig 2). Most studies were deemed to have low bias across several domains. However, a few studies lacked explicit descriptions of their selection criteria, one study lacked clear timing, and three studies lacked clear patient selection (Fig 2, S3 Appendix).

### Characteristics of included studies

Table 1 displays the characteristics of the eleven studies. All selected studies were published between 2021 and 2025 and used US, MRI, or CT. The sample sizes ranged from 20 to 12,534 participants. One study used MRI, seven studies used US, one study used CT, and two studies evaluated more than one imaging modality. AI techniques included convolutional neural networks (CNN), Sooty Tern Optimization, Deep R-Belief Neural Network, and RetinaNet. Six studies were prospective and five were retrospective.

**Table 1:** Table with the summary of articles.

Study	Year	Title	Journal	Country	Study Design	Data Set Size (n = patients)	Imaging Modality	Objective and AI Technique	Site	Images were evaluated by
Kainz et al.	2021	Non-invasive diagnosis of deep vein thrombosis from ultrasound imaging with machine learning	NPJ Digit Medicine	United Kingdom & Germany	Prospective	83	US	Using deep learning framework to evaluate US videos to aid in DVT diagnosis	External iliac vein Greater saphenous vein Common femoral vein Superficial femoral vein Deep femoral vein Popliteal vein	Radiologist
Sun et al.	2021	Deep learning for accurate segmentation of venous thrombus from black-blood Magnetic Resonance Images: A multicenter study	Biomed Research International	China	Retrospective	110	Black-blood MRI	Using deep learning network to segment venous thrombosis	Inferior vena cava Common iliac vein Internal iliac vein External iliac vein Common femoral vein Deep femoral vein Superficial femoral vein Popliteal vein Anterior tibial vein Posterior tibial vein Fibular vein Great saphenous vein Small saphenous vein	2 radiologists
Nakayama et al.	2023	Deep learning-based classification of adequate sonographic images for self-diagnosing deep vein thrombosis	PLOS One	Japan	Prospective	20	US	Using ResNet101 to classify images of popliteal vein for DVT diagnosis	Popliteal vein	First author and the sonographer (6^th^ author)
Seo et al.	2023	Artificial intelligence-based iliofemoral deep venous thrombosis detection using a clinical approach	Scientific Reports	South Korea	Retrospective	190	CT angiography	Using convolutional neural network based RetinaNet to detect DVT on synthesized images	Iliac vein Common femoral vein	Radiologists
Oppenheimer et al.	2024	Remote expert DVT triaging of novice-user compression sonography with AI-guidance	Annals of Vascular Surgery	Germany	Prospective	70	US	Using machine-learning software to diagnose DVT	Common femoral vein Superficial and Profunda femoral vein Popliteal vein Proximal trifurcation of the distal veins	5 external experts in a remote setting
Joseph et al.	2024	Deep Vein Net: Deep Vein Thrombosis Identification Via Sooty Tern Optimized Deep Learning Network	Revue Roumaine des Sciences Techniques –Electrotechnique et energetique	India	Retrospective	573	CT and MRI	Using deep learning CNN to diagnose DVT	Not specified	Radiologists from the Radiological Society of North America (RSNA) & the Society of Thoracic Radiology (STR)
Nothnagel & Aslam	2024	Evaluating the benefits of machine learning for diagnosing deep vein thrombosis compared with gold standard ultrasound: a feasibility study	BJGP Open	United Kingdom	Prospective	91	US	Using ThinkSono to guide diagnosis of DVT	Not specified	Qualified physician
Arun et al.	2025	Deep vein thrombosis detection via combination of neural networks	Biomedical Signal Processing and Control	India	Retrospective	12,534	US; additional comparative evaluation performed on CT and MRI datasets	Using Deep R-Belief Network to diagnose DVT	--	No human interpretation
Curry et al.	2025	Multicenter double-blind study evaluating AI-driven detection of proximal deep vein thrombosis	NEJM AI	United Kingdom	Prospective	414	US	Using AutoDVT to assist in the diagnosis of DVT	Common femoral and popliteal vein	Qualified clinician > 1 year of experience in diagnosing DVT by US
Speranza et al.	2025	Value of clinical review for AI-guided deep vein thrombosis diagnosis with ultrasound imaging by non-expert operators	NPJ Digital Medicine	United Kingdom	Retrospective	381	Point-of-Care US	Using ThinkSono to guide diagnosis of DVT	Common femoral and popliteal vein	Emergency Medicine (EM physicians and radiologists
Avgerinos et al.	2025	Novel AI guided non-expert compression ultrasound DVT diagnostic pathway may reduce vascular laboratory venous testing	European Journal of Vascular and Endovascular Surgery	Greece	Prospective	53	US	Using ThinkSono, to guide diagnosis of DVT	Two compressions in each area (common femoral vein, femoral and profunda femoral vein) and the popliteal fossa (popliteal vein, proximal trifurcation of the distal veins).	Radiologist

### Diagnostic performance of AI models by imaging modality

#### US-based models.

Kainz et al. (2021) conducted a prospective, multicenter study to evaluate a deep learning framework designed to use compression US to detect lower-extremity DVT. The datasets included US video loops collected from two sites using two different devices, ensuring heterogeneity across acquisition sources [29]. The model architecture integrated vessel segmentation, anatomical landmark detection, and compressibility classification into a single pipeline to assess vein patency. Internal and external validation were both performed, with external testing conducted across sites and devices [29]. Their model achieved a sensitivity between 82% and 96%, a specificity between 70% and 82%, and an accuracy between 75% and 83% [29]. This system was designed to guide non-specialists, with radiologists providing the ground truth labels [29]. An additional feature of this work was the evaluation of cost-effectiveness, demonstrating the authors’ emphasis on clinical integration [29].

Nakayama et al. (2023) conducted a prospective study using their ResNet101 on both stationary and portable ultrasound images of the popliteal vein. Instead of identifying the thrombi directly, their deep learning model was developed to determine whether images were of sufficient diagnostic quality to allow reliable assessment of DVT [33]. By automatically categorizing the images into “Satisfactory,” “Moderately Satisfactory,” and “Unsatisfactory” groups, the algorithm assessed the quality of image acquisition [33]. The team also reported comparable accuracies of 76% and 72% for stationary and portable ultrasound, respectively [33].

Oppenheimer et al. (2024) conducted a prospective study across two university hospitals to evaluate an AI-assisted US system integrated within a telemedicine framework for the diagnosis of DVT [34]. It provided novice operators real-time feedback on venous compressibility at the groin and popliteal fossa. The scans were then reviewed by remote specialists [34]. The Auto-DVT model demonstrated 100% sensitivity and 95% specificity [34].

Nothnagel and Aslam (2024) performed a prospective clinical evaluation of the ThinkSono AI-guided US. The algorithm utilized a CNN to analyze US in real time, providing acquisition guidance and classification of venous compressibility [35]. This work demonstrated a point-of-care implementation, in which a physician used the AI tool to acquire and interpret US images [35]. The reported diagnostic performance was 100% sensitivity and 90–92% specificity [35].

Curry et al. (2025) conducted a double-blind, multicenter, prospective trial of AutoDVT, a handheld compression US device used to diagnose DVT at the common femoral and popliteal veins [36]. The model combined CNN optimized for vessel recognition with algorithms that actively assessed probe positioning, vein centering, and compressibility during scanning [36]. The model demonstrated a sensitivity of 68% and a specificity of 80% for the detection of proximal DVT [36].

Avgerinos et al. (2025) conducted a prospective study to evaluate the ThinkSono AI-guided US platform for bedside detection of lower-extremity DVT [37]. Similar to the ThinkSono used in other studies, this model provided real-time prompts to non-expert operators to ensure correct probe positioning and adequate compression. Image adequacy was assessed using the American College of Emergency Physicians (ACEP) quality scoring [37]. This model achieved 100% sensitivity and 95% specificity [37].

Speranza et al. (2025) conducted a retrospective study evaluating the ThinkSono-guided point-of-care US. The study included both radiologists and emergency medicine physicians as operators, who performed compression ultrasound at the common femoral and popliteal veins with real-time AI guidance [38]. The reported diagnostic performance had a sensitivity of 90–98% and a specificity of 74–100% [38]. While ultrasound-based models dominate the literature, MRI studies provided insight into higher-resolution thrombus characterization [30].

#### MRI-based models.

Sun et al. (2021) retrospectively evaluated a deep learning segmentation model designed for the detection of lower extremity DVT on black-blood MRI. Its architecture of a 3D U-shaped CNN with a generative adversarial network (GAN) framework automatically segmented venous thrombi [30]. It was validated on multicenter datasets using sequences like DANTE-SPACE and DANTE-FLASH [30]. The team noted high performance in their deep learning model on black blood MRI, with a sensitivity ranging from 93% to 95%, a specificity between 92% and 97%, and an accuracy between 94% and 96% [30].

#### CT-based models.

Seo et al. (2023) conducted a retrospective study and developed a RetinaNet-based object detection framework with different backbones (ResNet50, ResNet152) to detect lower limb DVT on CT venography. The authors generated synthesized image patches with a three-slice input (one image, one image above, and one image below) to provide better anatomical context [15]. This study combined the detection of thrombosis with a focus on data preparation [15]. The reported diagnostic performance showed an average sensitivity of 83.3% [15]. Beyond single-modality approaches, some studies explored hybrid architectures to leverage complementary imaging strengths [39,40].

#### Studies evaluated across multiple imaging datasets.

Joseph et al. (2024) conducted a retrospective study and presented a deep learning framework termed “Deep Vein Net,” which integrated a CNN with a Sooty Tern Optimization (STO) algorithm. The excellent exploration ability and convergence speed of this metaheuristic optimization method mimic the foraging behavior of sooty terns [40]. In this application, the STO algorithm was used to adjust the hyperparameters of the CNN, thereby enhancing its ability to identify subtle spatial features across several MRI slices [40]. The reported diagnostic performance included a specificity of 96%, an accuracy of 96%, and a precision of 97% [40].

Arun et al. (2025) created the Deep R-Belief Neural Network, which was built from stacked Boltzmann machines. This approach enabled unsupervised pre-training, which was then followed by supervised fine-tuning, allowing the model to capture complex feature representations [39]. The network was evaluated across heterogeneous imaging datasets, allowing assessment of performance on ultrasound, CT, and MRI datasets independently. The algorithm was also trained on more than 12,000 publicly available datasets [39]. Their model achieved a specificity of 97%, an accuracy of 98.9%, and a precision of 97.5% [39].

## Discussion

This scoping review incorporated eleven studies published between 2021 and May 2025 on the use of AI for the imaging diagnosis of DVT. The results highlight both the potential and the limitations of AI in the field of radiology. US-based approaches such as ThinkSono and AutoDVT demonstrated high clinical relevance by targeting real-time bedside evaluation by non-expert operators [35,36,38]. MRI-based deep learning segmentation models and CT-based approaches both illustrated strong performance metrics [30]. Finally, studies evaluating multiple imaging datasets demonstrated the potential of AI approaches across different imaging sources [39,40]. Collectively, these findings suggest that AI can augment, but not yet replace, traditional imaging interpretation.

### Strengths and limitations

Strengths of this scoping review include a comprehensive literature search across seven major databases, ensuring the review’s completeness. Two independent reviewers and the use of the QUADAS-2 tool reduced selection bias and enhanced the study’s reliability. The studies in this review employed diverse AI methods, such as RetinaNet for small-object detection, Deep R-Belief Networks for separate evaluation across different imaging datasets, and Sooty Tern Optimization for exploration and convergence speed [15,39,40]. Each algorithm has a unique set of strengths and weaknesses.

Among all imaging modalities, US remains the most widely studied platform for AI-driven DVT detection, reflecting its role as the first-line imaging modality [11,13]. Several recent studies highlight both the promise and the variability of AI-guided ultrasound approaches [11,13].

#### US-based models.

AutoDVT from Kainz et al. (2021) demonstrated several strengths. It was clinically relevant for bedside use by non-experts and introduced technological innovation by combining vessel segmentation, landmark detection, and compressibility assessment into a single model [29]. The study also included external validation across two sites and devices, as well as cost-effectiveness analyses [29]. However, the authors also recognized several limitations. The authors acknowledged the risk of domain shift, a phenomenon in which the algorithm’s performance deteriorates when the dataset is applied to unfamiliar US devices [29]. Furthermore, the modest positive predictive values (65–89%) indicated that positive results would require expert review before diagnosis [29].

Nakayama et al. (2023) and their model exhibited comparable performance across both portable and stationary US, indicating adaptability in various healthcare settings. A key strength was its focus on image quality and the use of heatmaps to enhance interpretability [33]. The study had several limitations. The sample size was small (n = 20) and reported accuracies were modest (72–76%) [33]. The model lacked external validation and was limited to the popliteal vein, leaving performance at other sites untested. The intermediate category also showed higher misclassification rates, which could potentially lower its real-world applicability [33]. Overall, this study illustrates both the potential and the limitations of applying AI-assisted compressibility assessment to portable US. Further optimization and multicenter evaluation are needed before clinical deployment [33].

Oppenheimer et al. (2024) and their AI model provided novice operators with real-time feedback on venous compressibility. A strength of the study was its focus on clinical integration, combining AI guidance with telemedicine expertise [34]. The tool demonstrated high sensitivity (100%), suggesting promise as a frontline triage tool. Its specificity (95%) indicated fewer false positives compared to other US-based AI systems [34]. The model also prompted users to apply adequate compressions when scanning. However, the algorithm was trained only on two-point compression, leaving calf and iliac thromboses undetectable [34]. The AI tool was also only tested on a small sample size of 70. The authors emphasized the need for larger, prospective validation across diverse settings to establish applicability [34]. AutoDVT demonstrates promise as a rapid triage adjunct, but broader validation across diverse populations and US devices is necessary before clinical adoption [34].

Nothnagel and Aslam (2024) and their ThinkSono Guidance platform enabled non-experts to obtain adequate compression images [35]. A strength of this study was its focus on real-time image acquisition. The American College of Emergency Physicians (ACEP) quality score added objectivity and transparency to the evaluation process by grading images [35]. The scans could also be uploaded for independent expert review, improving diagnostic safety in the workflow [35]. The model also achieved near-perfect sensitivity, which is valuable for minimizing missed DVT cases, while maintaining specificity around 90%. However, this tool had a few limitations [35]. It was limited to a three-region proximal scan and did not evaluate the calves. The sample size was modest, and external testing was not reported, limiting generalizability [35]. Nevertheless, these findings strengthen the case for AI as a supportive triage tool in bedside US [35].

Curry et al. (2025) assessed AutoDVT, an AI system engineered to deliver real-time guidance and assessment of venous compressibility on handheld US [36]. The tool’s strength lies in its design to actively direct probe positioning and maintain reproducibility across operators. It could finish a targeted scan in as short as four minutes [36]. However, the algorithm demonstrated important limitations. It demonstrated modest diagnostic performance with a sensitivity of only 68% and a specificity of 80%, limiting its utility as a standalone tool [36]. Its diagnostic scope was restricted to the femoral and popliteal veins, and sensitivity was modest at 68%. The model was also only trained and validated on a single handheld device, which raised concerns about its generalizability [36]. Further optimization and broader validation across devices are needed before clinical integration [36].

The ThinkSono from Speranza et al. (2025) also directed novice operators through two-point compression scans, giving feedback on probe position, vein centering, and compression adequacy [38]. It had a decent sample size of 381 patients across 11 hospitals, demonstrating robustness in its results. However, the algorithm remained constrained to a two-point compression protocol, with calf and iliac thromboses unaddressed [38]. Inter-observer agreement for image quality was also low, and the system is not autonomous, instead requiring clinician input to establish a diagnosis [38].

Avgerinos et al. (2025) provided important prospective evidence for the clinical utility of ThinkSono in real-world practice. ThinkSono demonstrated high diagnostic accuracy. All scans achieved an American College of Emergency Physicians (ACEP) quality score of 3 or more, indicating the adequacy of the images collected [37]. The AI tool could complete a scan within 7 minutes on average, increasing efficiency. However, its limitations included limited anatomical scope, as the tool was restricted to the femoral and popliteal vein compression, excluding potential calf or iliac DVT [37]. Furthermore, the system required expert input, limiting its role as a fully autonomous diagnostic tool. Lastly, it was tested on a small sample size of 53 and included few DVT cases [37]. Overall, this study highlighted the feasibility and efficiency of AI-guided US for lower-extremity DVT but reinforced the need for larger multicenter trials to confirm generalizability [37].

While US-based AI models were the most extensively studied and clinically feasible, MRI-based approaches provided an opportunity to push diagnostic performance even higher, albeit with trade-offs in cost and accessibility [30].

#### MRI-based models.

Sun et al. (2021) used a 3D U-shaped segmentation model within a GAN framework to differentiate thrombus from tissues exhibiting similar intensity and morphology on MRI [30]. One strength of this work was its focus on pixel-level segmentation to differentiate thrombus from adjacent tissue with similar morphology. The model was validated on DANTE-SPACE and DANTE-FLASH sequences from three different centers [30]. Performance was strong and outperformed state-of-the-art models such as 3D U-Net, V-Net, 3D nnU-Net, and Cascade nnU-Net [30]. However, the need for extensive preprocessing steps such as resampling, normalization, and data augmentation made clinical implementation computationally demanding. Additionally, the model was not prospectively tested in real-world clinical workflows, and its performance across diverse MRI vendors or protocols was indeterminate [30]. Furthermore, MRI’s high cost and limited availability reduce immediate applicability in routine DVT evaluation, which more often relies on US. Overall, Sun et al. (2021) provided compelling evidence, but larger multicenter trials are needed before MRI-based AI models can be translated into clinical practice [30].

In contrast to MRI, which offers near-perfect diagnostic metrics but limited clinical scalability, CT-based AI studies remain relatively sparse and exploratory, highlighting both the feasibility and current gaps in this domain [15].

#### CT-based models.

The RetinaNet model from Seo et al. (2023) synthesized three consecutive CT slices to mimic how radiologists assess DVT across multiple images, improving diagnostic accuracy [15]. RetinaNet’s one-stage architecture performed detection and classification concurrently, making it more computationally efficient. Furthermore, the authors addressed data quality by excluding cases with significant artifacts to ensure the reliability of the training dataset. However, the authors noted a few limitations [15]. The AI model was restricted to iliofemoral veins, and clinically important calf veins were excluded. The sample size was modest, raising concerns about generalizability. The model was also not externally validated [15].

Beyond single-modality applications, frameworks using different imaging modalities may represent the next frontier, aiming to leverage complementary strengths across imaging techniques to achieve even greater diagnostic robustness [39,40].

#### Studies evaluated across multiple imaging datasets.

Joseph et al. (2024) employed “Deep Vein Net,” a hybrid algorithm that integrated discrete waveform pre-processing, Sooty Tern Optimization for feature selection, and a fuzzy Extreme Learning Machine classifier [40]. The model reported outstanding accuracy across CT and MRI, surpassing deep learning networks including Google Net, Ghost Net, and AlexNet [40]. However, several limitations were present. The study was retrospective and lacked external validation. Furthermore, its restriction to CT and MRI, which are not first-line modalities for DVT diagnosis, limited its immediate applicability to clinical workflows [39]. Overall, Joseph et al. (2024) provided a technically sophisticated model, but its clinical utility remains uncertain until validated prospectively in a diverse population [40].

Arun et al. (2025) and their model combined RegNet for feature extraction with Deep Belief classifier. It had high diagnostic performance, outperforming models such as CNN, SESARF, and XGBoost [39]. Although CT, MRI and ultrasound datasets were evaluated separately for comparative analysis, the proposed framework focused primarily on duplex ultrasound-based DVT classification rather than true multimodal fusion. No prospective testing or external validation was reported, raising concerns about generalizability [40]

Overall, these modality-specific findings suggest that ultrasound-based AI models are closest to clinical translation, MRI-based models highlight the technical frontier of accuracy, CT-based models remain exploratory, and approaches incorporating multiple imaging datasets represent an emerging but unvalidated future direction [15,29,30,33–40].

### Path to clinical implementation

Radiologists have traditionally played a key role in diagnosing DVT through image interpretation. The sensitivity and specificity for DVT detection are 94% and 97%, respectively, when performed and interpreted by specialized radiologists [29]. Some of the AI models in this review have achieved similar or better results.

Although AI models demonstrate encouraging results for AI in DVT diagnosis, translation into clinical practice requires overcoming several barriers. First, external validation across diverse populations and imaging platforms is necessary to ensure generalizability [41]. Second, integration with clinical factors such as risk factors, D-dimer results, and comorbidities could provide enhanced diagnostic support [42,43]. Third, institutional regulatory approval and clinical acceptance will depend on transparency and prospective trials [44]. Fourth, cost-effective analysis will also be required to evaluate whether AI can shorten diagnostic time, reduce time to treatment, and lower healthcare expenditures [29].

### Future directions

Future research should prioritize multicenter validation and incorporate organizations from various geographical locations and healthcare contexts to enhance the global utility and generalizability of AI models [41]. Furthermore, to facilitate stratified subgroup analysis, larger sample sizes are needed to ensure AI tools perform reliably across different patient populations and clinical scenarios. Biased outputs may exacerbate healthcare disparities [41].

More work can be done on prospective validation in real-world healthcare settings by comparing AI performance with that of radiologists [45]. Prospective implementation studies in emergency care and primary care will be critical to determine how AI can complement radiologists and extend diagnostic capacity for non-specialist operators [45].

## Conclusion

This scoping review shows that AI models may enhance the imaging diagnosis of DVT. Promising diagnostic performance was demonstrated across US, CT, and MRI. Notably, ThinkSono and AutoDVT highlight the feasibility of real-time bedside support for non-expert operators, while MRI and CT models could detect thrombi with high precision [15,30,35–38]. These findings suggest that AI can enhance workflows and increase accessibility to early DVT diagnosis.

Nonetheless, the evidence on this topic remains nascent. Most models were trained on relatively small or single-center datasets and tested under controlled conditions. Risks of domain shift and the lack of prospective multicenter validation limit the generalizability of current results [29,41]. Furthermore, positive predictive values remain modest for some studies, requiring confirmatory expert review [29,41].

Current evidence suggests that AI is best suited as a supportive adjunct rather than a replacement for expert interpretation in DVT diagnosis. Future research should emphasize external validation across diverse populations and imaging modalities [41]. Efforts should also focus on integrating clinical variables and developing explainable models to foster clinician trust. Lastly, large studies evaluating workflow efficiency and cost-effectiveness will be essential for translating AI from concept to routine practice [29,41].

## Supporting information

S1 Appendix Preferred Reporting Items for Systematic reviews and Meta-Analyses extension for Scoping Reviews (PRISMA-ScR) Checklist. (DOCX)

S2 Appendix Search Strategy Across 7 Major Online Databases. (DOCX)

S3 Appendix QUADAS-2, Quality Assessment of Diagnostic Accuracy of Studies. (DOC)

S4 Appendix Fundamentals of Artificial Intelligence (AI). (DOCX)

S1 Table Sensitivity, specificity, accuracy, and precision of AI models on CT, US, and MRI. (DOCX)
