# Supplementary material for: Artificial intelligence in the diagnosis of deep vein thrombosis: A scoping review
Source: PLoS One. 2026 Jun 22;21(6):e0351558. doi: 10.1371/journal.pone.0351558 (PMC13286142; doi:10.1371/journal.pone.0351558)
Supplement: S2 Appendix — (DOCX) [file pone.0351558.s002.docx]

**S2. Search Strategy Across 7 Major Online Databases**

| No | **Databases** | **Search Strategy** | Hits |
| --- | --- | --- | --- |
| 1 | Pubmed | **(((deep AND (vein[tiab] OR venous[tiab]) AND (thrombos*[tiab] OR thrombi*[tiab] OR thrombus[tiab] OR thromboembol*[tiab])) OR deep-vein[tiab] OR deep-venous[tiab]) OR DVT[Title/Abstract] OR ((‘‘Venous Thrombosis’’[Mesh]))) OR (((‘‘Venous Thromboembolism’’[Mesh]))) AND ("Artificial Intelligence"[tiab] OR "AI"[tiab] OR "deep learning"[tiab] OR "machine learning"[tiab] OR (Artificial Intelligence[MeSH Terms]))** | 569 |
| 2 | CINAHL | S1 TI ((((deep AND (vein OR venous) AND (thrombos*OR thrombi* OR thrombus OR thromboembol*)) OR deep-vein OR deep-venous) OR DVT) OR AB ((((deep AND (vein OR venous) AND (thrombos*OR thrombi* OR thrombus OR thromboembol*)) OR deep-vein OR deep-venous) OR DVT)  S2 TI ( ("Artificial Intelligence" OR "AI" OR "deep learning" OR "machine learning") OR AB (("Artificial Intelligence" OR "AI" OR "deep learning" OR "machine learning")  S3 (MH "Venous Thrombosis")  S4 (MM "Venous Thromboembolism")  S5 (MH "Artificial Intelligence+")  S6 (S1 OR S3 OR S4)  S7 (S2 OR S5)  S8 (S6 AND S7) | 142 |
| 3 | Web of Science | 7 #5 AND #6  6 #3 OR #4  5 #1 OR #2  4 AB=((("Artificial Intelligence" OR "AI" OR "deep learning" OR "machine learning")))  3 TI=((("Artificial Intelligence" OR "AI" OR "deep learning" OR "machine learning")))  2 AB=((((((((deep AND (vein OR venous) AND (thrombos*OR thrombi* OR thrombus OR thromboembol*)) OR deep-veinOR deep-venous) OR DVT)))))  1 TI=((((((((deep AND (vein OR venous) AND (thrombos*OR thrombi* OR thrombus OR thromboembol*)) OR deep-veinOR deep-venous) OR DVT)))))) | 226 |
| 4 | Cochrane | ID Search Hits  #1 (deep AND (vein OR venous) AND (thrombos*OR thrombi* OR thrombus OR thromboembol*) OR deep-vein OR deep-venous) OR DVT  #2 ("Artificial Intelligence" OR "AI" OR "deep learning" OR "machine learning")  #3 MeSH descriptor: [Venous Thromboembolism] this term only  #4 MeSH descriptor: [Venous Thrombosis] explode all trees  #5 MeSH descriptor: [Artificial Intelligence] explode all trees  #6 #1 or #3 or #4  #7 #2 or #5  #8 #6 and #7 | 105 |
| 5 | Scopus | ((((deep AND (vein OR venous) AND (thrombos*or AND thrombi* OR thrombus OR thromboembol*)) OR deep-vein OR deep-venous) OR DVT))  ("Artificial Intelligence" OR "AI" OR "deep learning" OR "machine learning")  Article and Review | 529 |
| 6 | Google scholar | ((((deep AND (vein OR venous) AND (thrombos*OR thrombi* OR thrombus OR thromboembol*)) OR deep-vein OR deep-venous) OR DVT) AND (("Artificial Intelligence" OR "AI" OR "deep learning" OR "machine learning") | 362 |
| 7 | Proquest | S3  [S1] AND [S2]  S2  abstract("Artificial Intelligence" OR AI OR "deep learning" OR "machine learning") OR title("Artificial Intelligence" OR AI OR "deep learning" OR "machine learning")  S1  abstract(((((deep AND (vein OR venous) AND (thrombos*or AND thrombi* OR thrombus OR thromboembol*)) OR deep-vein OR deep-venous) OR dvt))) OR title(((((deep AND (vein OR venous) AND (thrombos*or AND thrombi* OR thrombus OR thromboembol*)) OR deep-vein OR deep-venous) OR dvt ))) | 152 |
