# Supplementary material for: Artificial intelligence in the diagnosis of deep vein thrombosis: A scoping review
Source: PLoS One. 2026 Jun 22;21(6):e0351558. doi: 10.1371/journal.pone.0351558 (PMC13286142; doi:10.1371/journal.pone.0351558)
Supplement: S3 Appendix — (DOC) [file pone.0351558.s003.doc]

**S3.** **QUADAS-2, Quality Assessment of Diagnostic Accuracy of Studies**

Sun et al 2021

QUADAS-2 tool: Risk of bias and applicability judgments

| **Domain 1: Patient selection** | |
| --- | --- |
| 1. **Risk of bias** |  |
| **Describe methods of patient selection:**  110 subjects are obtained from three different centers using two different MRI blackblood techniques were selected | |
| - **Was a consecutive or random sample of patients enrolled?** | Yes/No/Unclear |
| - **Was a case-control design avoided?** | Yes/No/Unclear |
| - **Did the study avoid inappropriate exclusions?** | Yes/No/Unclear |
| **Could the selection of patients have introduced bias?** | RISK: LOW/HIGH/UNCLEAR |
| 1. **Concerns regarding applicability** |  |
| **Describe included patients (prior testing, presentation, intended use of index test and setting):**  The ground truth of thrombus lesions is manually contoured with the consensus between two experienced radiologists on the BTI images. | |
| **Is there concern that the included patients do not match the review question?** | CONCERN: LOW/HIGH/UNCLEAR |
| **Domain 2: Index test(s) *(if more than 1 index test was used, please complete for each test)*** | |
| 1. **Risk of bias** |  |
| **Describe the index test and how it was conducted and interpreted:**  The proposed deep learning network for automatic venous thrombus segmentation from BTI images with high accuracy and reliability. This network was tested and externally tested by subjects from 3 different centers. | |
| - **Were the index test results interpreted without knowledge of the results of the reference standard?** | Yes/No/Unclear |
| - **If a threshold was used, was it pre-specified?** | Yes/No/Unclear |
| **Could the conduct or interpretation of the index test have introduced bias?** | RISK: LOW/HIGH/UNCLEAR |
| 1. **Concerns regarding applicability** |  |
| **Is there concern that the index test, its conduct, or interpretation differ from the review question?** | CONCERN: LOW/HIGH/UNCLEAR |
| **Domain 3: Reference standard** | |
| 1. **Risk of bias** |  |
| **Describe the reference standard and how it was conducted and interpreted:**  The ground truth of thrombus lesions is manually contoured with the consensus between two experienced radiologists on the BTI images. | |
| - **Is the reference standard likely to correctly classify the target condition?** | Yes/No/Unclear |
| - **Were the reference standard results interpreted without knowledge of the results of the index test?** | Yes/No/Unclear |
| **Could the reference standard, its conduct, or its interpretation have introduced bias?** | RISK: LOW/HIGH/UNCLEAR |
| 1. **Concerns regarding applicability** |  |
| **Is there concern that the target condition as defined by the reference standard does not match the review question?** | CONCERN: LOW/HIGH/UNCLEAR |
| **Domain 4: Flow and timing** | |
| 1. **Risk of bias** |  |
| **Describe any patients who did not receive the index test(s) and/or reference standard or who were excluded from the 2x2 table (refer to flow diagram):**  None  **Describe the time interval and any interventions between index test(s) and reference standard:**  Unclear | |
| - **Was there an appropriate interval between index test(s) and reference standard?** | Yes/No/Unclear |
| - **Did all patients receive a reference standard?** | Yes/No/Unclear |
| - **Did patients receive the same reference standard?** | Yes/No/Unclear |
| - **Were all patients included in the analysis?** | Yes/No/Unclear |
| **Could the patient flow have introduced bias?** | RISK: LOW/HIGH/UNCLEAR |

Kainz et al 2021

QUADAS-2 tool: Risk of bias and applicability judgments

| **Domain 1: Patient selection** | |
| --- | --- |
| 1. **Risk of bias** |  |
| **Describe methods of patient selection:**  The study was conducted in a clinical setting, specifically at an NHS DVT diagnostic clinic in Oxford, UK, and a German DVT clinic (124 patients) with symptoms of DVT. The index test, which involves the use of a deep learning algorithm to interpret ultrasound images, is intended for the non-specialist diagnosis of DVT at the point of care, aiming to aid non-specialists in detecting DVT more effectively. | |
| - **Was a consecutive or random sample of patients enrolled?** | Yes/No/Unclear |
| - **Was a case-control design avoided?** | Yes/No/Unclear |
| - **Did the study avoid inappropriate exclusions?** | Yes/No/Unclear |
| **Could the selection of patients have introduced bias?** | RISK: LOW/HIGH/UNCLEAR |
| 1. **Concerns regarding applicability** |  |
| **Describe included patients (prior testing, presentation, intended use of index test and setting):**  Prior Testing: Patients presenting with symptoms suggestive of DVT, likely having undergone initial clinical assessments or preliminary imaging as part of the standard diagnostic workflow for DVT.  Presentation: The patients were symptomatic, seeking diagnosis for suspected DVT at specialized DVT clinics. Intended Use of Index Test: The deep learning algorithm was intended to aid non-specialists in the accurate detection of DVT using ultrasound imaging at the point of care, thus enhancing diagnostic accessibility and potentially reducing specialist referral times.  Setting: The study was conducted in clinical settings, specifically in DVT clinics within NHS settings in the UK and a German clinic, which implies a typical healthcare facility environment equipped for specialized diagnostic procedures. | |
| **Is there concern that the included patients do not match the review question?** | CONCERN: LOW/HIGH/UNCLEAR |
| **Domain 2: Index test(s) *(if more than 1 index test was used, please complete for each test)*** | |
| 1. **Risk of bias** |  |
| **Describe the index test and how it was conducted and interpreted:**  The patients underwent ultrasound imaging of the legs using a Philips Lumify probe with screen recording software. Typical ultrasound scanning procedures adapted for use with the AI guidance system. | |
| - **Were the index test results interpreted without knowledge of the results of the reference standard?** | Yes/No/Unclear |
| - **If a threshold was used, was it pre-specified?** | Yes/No/Unclear |
| **Could the conduct or interpretation of the index test have introduced bias?** | RISK: LOW/HIGH/UNCLEAR |
| 1. **Concerns regarding applicability** |  |
| **Is there concern that the index test, its conduct, or interpretation differ from the review question?** | CONCERN: LOW/HIGH/UNCLEAR |
| **Domain 3: Reference standard** | |
| 1. **Risk of bias** |  |
| **Describe the reference standard and how it was conducted and interpreted:** | |
| - **Is the reference standard likely to correctly classify the target condition?** | Yes/No/Unclear |
| - **Were the reference standard results interpreted without knowledge of the results of the index test?** | Yes/No/Unclear |
| **Could the reference standard, its conduct, or its interpretation have introduced bias?** | RISK: LOW/HIGH/UNCLEAR |
| 1. **Concerns regarding applicability** |  |
| **Is there concern that the target condition as defined by the reference standard does not match the review question?** | CONCERN: LOW/HIGH/UNCLEAR |
| **Domain 4: Flow and timing** | |
| 1. **Risk of bias** |  |
| **Describe any patients who did not receive the index test(s) and/or reference standard or who were excluded from the 2x2 table (refer to flow diagram):**  None  **Describe the time interval and any interventions between index test(s) and reference standard:**  Not noted in the literature | |
| - **Was there an appropriate interval between index test(s) and reference standard?** | Yes/No/Unclear |
| - **Did all patients receive a reference standard?** | Yes/No/Unclear |
| - **Did patients receive the same reference standard?** | Yes/No/Unclear |
| - **Were all patients included in the analysis?** | Yes/No/Unclear |
| **Could the patient flow have introduced bias?** | RISK: LOW/HIGH/UNCLEAR |

Nakayama 2023 et al

QUADAS-2 tool: Risk of bias and applicability judgments

| **Domain 1: Patient selection** | |
| --- | --- |
| 1. **Risk of bias** |  |
| **Describe methods of patient selection:**  Study was conducted in Japan. The index test, a deep learning model (ResNet101), was intended for automatically classifying the adequacy of sonographic images of the popliteal vein. Healthy volunteers were recruited, there was no mention of symptomatic patients being recruited. | |
| - **Was a consecutive or random sample of patients enrolled?** | Yes/No/Unclear |
| - **Was a case-control design avoided?** | Yes/No/Unclear |
| - **Did the study avoid inappropriate exclusions?** | Yes/No/Unclear |
| **Could the selection of patients have introduced bias?** | RISK: LOW/HIGH/UNCLEAR |
| 1. **Concerns regarding applicability** |  |
| **Describe included patients (prior testing, presentation, intended use of index test and setting):**  Healthy patients | |
| **Is there concern that the included patients do not match the review question?** | CONCERN: LOW/HIGH/UNCLEAR |
| **Domain 2: Index test(s) *(if more than 1 index test was used, please complete for each test)*** | |
| 1. **Risk of bias** |  |
| **Describe the index test and how it was conducted and interpreted:**  The authors used a deep learning based classification system developed to determine whether sonographic images are adequate for diagnosing DVT | |
| - **Were the index test results interpreted without knowledge of the results of the reference standard?** | Yes/No/Unclear |
| - **If a threshold was used, was it pre-specified?** | Yes/No/Unclear |
| **Could the conduct or interpretation of the index test have introduced bias?** | RISK: LOW/HIGH/UNCLEAR |
| 1. **Concerns regarding applicability** |  |
| **Is there concern that the index test, its conduct, or interpretation differ from the review question?** | CONCERN: LOW/HIGH/UNCLEAR |
| **Domain 3: Reference standard** | |
| 1. **Risk of bias** |  |
| **Describe the reference standard and how it was conducted and interpreted:**  No clear reference standard as the healthy subjects are used to refine a pre-existing model.  Unclear what the reference standard is. | |
| - **Is the reference standard likely to correctly classify the target condition?** | Yes/No/Unclear |
| - **Were the reference standard results interpreted without knowledge of the results of the index test?** | Yes/No/Unclear |
| **Could the reference standard, its conduct, or its interpretation have introduced bias?** | RISK: LOW/HIGH/UNCLEAR |
| 1. **Concerns regarding applicability** |  |
| **Is there concern that the target condition as defined by the reference standard does not match the review question?** | CONCERN: LOW/HIGH/UNCLEAR |
| **Domain 4: Flow and timing** | |
| 1. **Risk of bias** |  |
| **Describe any patients who did not receive the index test(s) and/or reference standard or who were excluded from the 2x2 table (refer to flow diagram):**  None. All patients received the test  **Describe the time interval and any interventions between index test(s) and reference standard:**  None | |
| - **Was there an appropriate interval between index test(s) and reference standard?** | Yes/No/Unclear |
| - **Did all patients receive a reference standard?** | Yes/No/Unclear |
| - **Did patients receive the same reference standard?** | Yes/No/Unclear |
| - **Were all patients included in the analysis?** | Yes/No/Unclear |
| **Could the patient flow have introduced bias?** | RISK: LOW/HIGH/UNCLEAR |

Seo et al 2023

QUADAS-2 tool: Risk of bias and applicability judgments

| **Domain 1: Patient selection** | |
| --- | --- |
| 1. **Risk of bias** |  |
| **Describe methods of patient selection:**  Unknown presentation of the patients, patients who had a LDCTA were selected | |
| - **Was a consecutive or random sample of patients enrolled?** | Yes/No/Unclear |
| - **Was a case-control design avoided?** | Yes/No/Unclear |
| - **Did the study avoid inappropriate exclusions?** | Yes/No/Unclear |
| **Could the selection of patients have introduced bias?** | RISK: LOW/HIGH/UNCLEAR |
| 1. **Concerns regarding applicability** |  |
| **Describe included patients (prior testing, presentation, intended use of index test and setting):**  In accordance with the relevant guidelines and regulations in compliance with the Declaration of Helsinki. The picture archiving and communication system database was searched for LECTA examinations conducted at Gil Medical Center between January 2013 and December 2020, and 583 consecutive LECTA examinations were identified. When a patient underwent multiple LECTA examination sessions, only the first LECTA scan session of the patient was considered for this study. | |
| **Is there concern that the included patients do not match the review question?** | CONCERN: LOW/HIGH/UNCLEAR |
| **Domain 2: Index test(s) *(if more than 1 index test was used, please complete for each test)*** | |
| 1. **Risk of bias** |  |
| **Describe the index test and how it was conducted and interpreted:**  Use of the AI program was performed | |
| - **Were the index test results interpreted without knowledge of the results of the reference standard?** | Yes/No/Unclear |
| - **If a threshold was used, was it pre-specified?** | Yes/No/Unclear |
| **Could the conduct or interpretation of the index test have introduced bias?** | RISK: LOW/HIGH/UNCLEAR |
| 1. **Concerns regarding applicability** |  |
| **Is there concern that the index test, its conduct, or interpretation differ from the review question?** | CONCERN: LOW/HIGH/UNCLEAR |
| **Domain 3: Reference standard** | |
| 1. **Risk of bias** |  |
| **Describe the reference standard and how it was conducted and interpreted:**  The reference standard involves assessment by radiologists who diagnosed iliofemoral DVT. | |
| - **Is the reference standard likely to correctly classify the target condition?** | Yes/No/Unclear |
| - **Were the reference standard results interpreted without knowledge of the results of the index test?** | Yes/No/Unclear |
| **Could the reference standard, its conduct, or its interpretation have introduced bias?** | RISK: LOW/HIGH/UNCLEAR |
| 1. **Concerns regarding applicability** |  |
| **Is there concern that the target condition as defined by the reference standard does not match the review question?** | CONCERN: LOW/HIGH/UNCLEAR |
| **Domain 4: Flow and timing** | |
| 1. **Risk of bias** |  |
| **Describe any patients who did not receive the index test(s) and/or reference standard or who were excluded from the 2x2 table (refer to flow diagram):**  The cases without a detailed mention of the presence or absence of iliofemoral DVT in the radiologic report were excluded.  **Describe the time interval and any interventions between index test(s) and reference standard:** Unknown | |
| - **Was there an appropriate interval between index test(s) and reference standard?** | Yes/No/Unclear |
| - **Did all patients receive a reference standard?** | Yes/No/Unclear |
| - **Did patients receive the same reference standard?** | Yes/No/Unclear |
| - **Were all patients included in the analysis?** | Yes/No/Unclear |
| **Could the patient flow have introduced bias?** | RISK: LOW/HIGH/UNCLEAR |

Joseph et al 2024

QUADAS-2 tool: Risk of bias and applicability judgment

| **Domain 1: Patient selection** | |
| --- | --- |
| 1. **Risk of bias** |  |
| **Describe methods of patient selection:**  The PE diagnosis dataset resulted from a collaboration between the Radiological Society of North America (RSNA) and the Society of Thoracic Radiology (STR). The employed method was CT angiography of the pulmonary arteries, also known as computed tomography of the chest images (CTPA). For this research, we focused on utilizing two labels (Acute PE and Chronic PE) with binary classification for annotation. A total of 9446 CTPA exams were conducted, with 7279 sets | |
| - **Was a consecutive or random sample of patients enrolled?** | Yes/No/Unclear |
| - **Was a case-control design avoided?** | Yes/No/Unclear |
| - **Did the study avoid inappropriate exclusions?** | Yes/No/Unclear |
| **Could the selection of patients have introduced bias?** | RISK: LOW/HIGH/UNCLEAR |
| 1. **Concerns regarding applicability** |  |
| **Describe included patients (prior testing, presentation, intended use of index test and setting):**  It it unclear whether the patients match the review question as it was not clearly stated | |
| **Is there concern that the included patients do not match the review question?** | CONCERN: LOW/HIGH/UNCLEAR |
| **Domain 2: Index test(s) *(if more than 1 index test was used, please complete for each test)*** | |
| 1. **Risk of bias** |  |
| **Describe the index test and how it was conducted and interpreted:**  Deep vein net to identify DVT on CT and MRI | |
| - **Were the index test results interpreted without knowledge of the results of the reference standard?** | Yes/No/Unclear |
| - **If a threshold was used, was it pre-specified?** | Yes/No/Unclear |
| **Could the conduct or interpretation of the index test have introduced bias?** | RISK: LOW/HIGH/UNCLEAR |
| 1. **Concerns regarding applicability** |  |
| **Is there concern that the index test, its conduct, or interpretation differ from the review question?** | CONCERN: LOW/HIGH/UNCLEAR |
| **Domain 3: Reference standard** | |
| 1. **Risk of bias** |  |
| **Describe the reference standard and how it was conducted and interpreted:**  The Joseph et al. paper does not specify a traditional clinical reference standard explicitly or the use of DVT US as the reference standard. It does however compare it’s model with existing and traditional deep learning models. | |
| - **Is the reference standard likely to correctly classify the target condition?** | Yes/No/Unclear |
| - **Were the reference standard results interpreted without knowledge of the results of the index test?** | Yes/No/Unclear |
| **Could the reference standard, its conduct, or its interpretation have introduced bias?** | RISK: LOW/HIGH/UNCLEAR |
| 1. **Concerns regarding applicability** |  |
| **Is there concern that the target condition as defined by the reference standard does not match the review question?** | CONCERN: LOW/HIGH/UNCLEAR |
| **Domain 4: Flow and timing** | |
| 1. **Risk of bias** |  |
| **Describe any patients who did not receive the index test(s) and/or reference standard or who were excluded from the 2x2 table (refer to flow diagram):**  The PE diagnosis dataset resulted from a collaboration between the Radiological Society of North America (RSNA) and the Society of Thoracic Radiology (STR).  **Describe the time interval and any interventions between index test(s) and reference standard:**  Deep vein net to identify DVT on CT and MRI | |
| - **Was there an appropriate interval between index test(s) and reference standard?** | Yes/No/Unclear |
| - **Did all patients receive a reference standard?** | Yes/No/Unclear |
| - **Did patients receive the same reference standard?** | Yes/No/Unclear |
| - **Were all patients included in the analysis?** | Yes/No/Unclear |
| **Could the patient flow have introduced bias?** | RISK: LOW/HIGH/UNCLEAR |

Oppenheimer et al 2023

QUADAS-2 tool: Risk of bias and applicability judgments

| **Domain 1: Patient selection** | |
| --- | --- |
| 1. **Risk of bias** |  |
| **Describe methods of patient selection:**  Patients with clinical suspicion for DVT, recruited consecutively across two sites over 8 months. | |
| - **Was a consecutive or random sample of patients enrolled?** | Yes/No/Unclear |
| - **Was a case-control design avoided?** | Yes/No/Unclear |
| - **Did the study avoid inappropriate exclusions?** | Yes/No/Unclear |
| **Could the selection of patients have introduced bias?** | RISK: LOW/HIGH/UNCLEAR |
| 1. **Concerns regarding applicability** |  |
| **Describe included patients (prior testing, presentation, intended use of index test and setting):**  Patients with suspected lower extremity deep vein thrombosis (DVT) were recruited over an 8-month period from two hospitals in Germany and Greece. Recruited patients had no prior formal ultrasound prior to presentation. This study aimed to evaluate the use of AI-guided two-point compression ultrasound (AutoDVT), performed by novice healthcare providers using a mobile device, for triaging DVT. The reference standard was expert-performed duplex compression ultrasound to diagnose proximal DVT. | |
| **Is there concern that the included patients do not match the review question?** | CONCERN: LOW/HIGH/UNCLEAR |
| **Domain 2: Index test(s) *(if more than 1 index test was used, please complete for each test)*** | |
| 1. **Risk of bias** |  |
| **Describe the index test and how it was conducted and interpreted:**  AutoDVT guided the user to acquire 4 ultrasound clips (2 at the groin & 2 at the popliteal region), with real-time AI feedback. Clips were uploaded for blinded remote review. | |
| - **Were the index test results interpreted without knowledge of the results of the reference standard?** | Yes/No/Unclear |
| - **If a threshold was used, was it pre-specified?** | Yes/No/Unclear  (ACEP >=3 is adequate)  (Compressible/Incompressible was used as decision criteria) |
| **Could the conduct or interpretation of the index test have introduced bias?** | RISK: LOW/HIGH/UNCLEAR |
| 1. **Concerns regarding applicability** |  |
| **Is there concern that the index test, its conduct, or interpretation differ from the review question?** | CONCERN: LOW/HIGH/UNCLEAR |
| **Domain 3: Reference standard** | |
| 1. **Risk of bias** |  |
| **Describe the reference standard and how it was conducted and interpreted:**  The reference standard was formal duplex ultrasound using institutional protocols including doppler ultrasound and compression at multiple leg levels.  The performer was blinded to index test results. | |
| - **Is the reference standard likely to correctly classify the target condition?** | Yes/No/Unclear |
| - **Were the reference standard results interpreted without knowledge of the results of the index test?** | Yes/No/Unclear |
| **Could the reference standard, its conduct, or its interpretation have introduced bias?** | RISK: LOW/HIGH/UNCLEAR |
| 1. **Concerns regarding applicability** |  |
| **Is there concern that the target condition as defined by the reference standard does not match the review question?** | CONCERN: LOW/HIGH/UNCLEAR |
| **Domain 4: Flow and timing** | |
| 1. **Risk of bias** |  |
| **Describe any patients who did not receive the index test(s) and/or reference standard or who were excluded from the 2x2 table (refer to flow diagram):**  3 patients were excluded due to incomplete artificial intelligence (AI) scans  3 patients excluded because the protocol was not followed.  **Describe the time interval and any interventions between index test(s) and reference standard:**  AI-guided and formal scans were performed sequentially during the same clinical encounter. | |
| - **Was there an appropriate interval between index test(s) and reference standard?** | Yes/No/Unclear |
| - **Did all patients receive a reference standard?** | Yes/No/Unclear |
| - **Did patients receive the same reference standard?** | Yes/No/Unclear |
| - **Were all patients included in the analysis?** | Yes/No/Unclear |
| **Could the patient flow have introduced bias?** | RISK: LOW/HIGH/UNCLEAR |

Arun et al 2024

QUADAS-2 tool: Risk of bias and applicability judgments

| **Domain 1: Patient selection** | |
| --- | --- |
| 1. **Risk of bias** |  |
| **Describe methods of patient selection:**  Retrospective dataset from public sources. No prospective recruitment or random/consecutive selection described. | |
| - **Was a consecutive or random sample of patients enrolled?** | Yes/No/Unclear |
| - **Was a case-control design avoided?** | Yes/No/Unclear |
| - **Did the study avoid inappropriate exclusions?** | Yes/No/Unclear |
| **Could the selection of patients have introduced bias?** | RISK: LOW/HIGH/UNCLEAR |
| 1. **Concerns regarding applicability** |  |
| **Describe included patients (prior testing, presentation, intended use of index test and setting):**  This study utilized publicly available CT, MRI, and duplex ultrasound (DUS) images from patients with and without venous thromboembolism. The aim was to evaluate the diagnostic performance of a Deep R-Belief neural network for automated DVT classification. No formal reference standard was specified, and dataset labels were used as the basis for evaluating model performance. The target conditions included coronary thrombosis, pulmonary embolism, and venous thromboembolism, categorized by the AI model. | |
| **Is there concern that the included patients do not match the review question?** | CONCERN: LOW/HIGH/UNCLEAR |
| **Domain 2: Index test(s) *(if more than 1 index test was used, please complete for each test)*** | |
| 1. **Risk of bias** |  |
| **Describe the index test and how it was conducted and interpreted:**  The index test was a combination of RegNet Belief network (for feature extraction) and deep belief network (DBN) (for classification). The index text was conducted entirely via automated processing, no human interpretation step was involved. | |
| - **Were the index test results interpreted without knowledge of the results of the reference standard?** | Yes/No/Unclear |
| - **If a threshold was used, was it pre-specified?** | Yes/No/Unclear  (Classification labels were fixed)z |
| **Could the conduct or interpretation of the index test have introduced bias?** | RISK: LOW/HIGH/UNCLEAR |
| 1. **Concerns regarding applicability** |  |
| **Is there concern that the index test, its conduct, or interpretation differ from the review question?** | CONCERN: LOW/HIGH/UNCLEAR |
| **Domain 3: Reference standard** | |
| 1. **Risk of bias** |  |
| **Describe the reference standard and how it was conducted and interpreted:**  No gold-standard human expert diagnosis or clinical confirmation was described.  Datasets use pre-labelled categories from source databases. | |
| - **Is the reference standard likely to correctly classify the target condition?** | Yes/No/Unclear |
| - **Were the reference standard results interpreted without knowledge of the results of the index test?** | Yes/No/Unclear |
| **Could the reference standard, its conduct, or its interpretation have introduced bias?** | RISK: LOW/HIGH/UNCLEAR |
| 1. **Concerns regarding applicability** |  |
| **Is there concern that the target condition as defined by the reference standard does not match the review question?** | CONCERN: LOW/HIGH/UNCLEAR |
| **Domain 4: Flow and timing** | |
| 1. **Risk of bias** |  |
| **Describe any patients who did not receive the index test(s) and/or reference standard or who were excluded from the 2x2 table (refer to flow diagram):**  Not applicable as the dataset was from publicaly available dataset.  **Describe the time interval and any interventions between index test(s) and reference standard:**  Not applicable as data as from retrospective image analysis. | |
| - **Was there an appropriate interval between index test(s) and reference standard?** | Yes/No/Unclear/Not applicable |
| - **Did all patients receive a reference standard?** | Yes/No/Unclear/Not applicable |
| - **Did patients receive the same reference standard?** | Yes/No/Unclear |
| - **Were all patients included in the analysis?** | Yes/No/Unclear |
| **Could the patient flow have introduced bias?** | RISK: LOW/HIGH/UNCLEAR |

| **Was there an appropriate interval between index test(s) and reference standard?** | Yes/No/Unclear/Not applicable |
| --- | --- |
| - **Did all patients receive a reference standard?** | Yes/No/Unclear/Not applicable |
| - **Did patients receive the same reference standard?** | Yes/No/Unclear |
| - **Were all patients included in the analysis?** | Yes/No/Unclear |
| **Could the patient flow have introduced bias?** | RISK: LOW/HIGH/UNCLEAR |

Nothnagel & Aslam 2025

QUADAS-2 tool: Risk of bias and applicability judgments

| **Domain 1: Patient selection** | |
| --- | --- |
| 1. **Risk of bias** |  |
| **Describe methods of patient selection:**  The patients were recruited consecutively over 3.5 months at a Berlin hospital. The recruitment criteria required suspected DVT and need for ultrasound. | |
| - **Was a consecutive or random sample of patients enrolled?** | Yes/No/Unclear |
| - **Was a case-control design avoided?** | Yes/No/Unclear |
| - **Did the study avoid inappropriate exclusions?** | Yes/No/Unclear |
| **Could the selection of patients have introduced bias?** | RISK: LOW/HIGH/UNCLEAR |
| 1. **Concerns regarding applicability** |  |
| **Describe included patients (prior testing, presentation, intended use of index test and setting):**  Patients from primary care, inpatient wards, or scheduled appointments were recruited at a Berlin over 3.5 months. No prior formal imaging was required.  Index test was intended for triage in both primary and secondary care settings. | |
| **Is there concern that the included patients do not match the review question?** | CONCERN: LOW/HIGH/UNCLEAR |
| **Domain 2: Index test(s) *(if more than 1 index test was used, please complete for each test)*** | |
| 1. **Risk of bias** |  |
| **Describe the index test and how it was conducted and interpreted:**  AI-guided POCUS was performed by non-specialists after 1 hour of training using a mobile probe and application. Images were uploaded and evaluated remotely by five blinded experts. | |
| - **Were the index test results interpreted without knowledge of the results of the reference standard?** | Yes/No/Unclear |
| - **If a threshold was used, was it pre-specified?** | Yes/No/Unclear  (ACEP > =3 = sufficient quality) |
| **Could the conduct or interpretation of the index test have introduced bias?** | RISK: LOW/HIGH/UNCLEAR |
| 1. **Concerns regarding applicability** |  |
| **Is there concern that the index test, its conduct, or interpretation differ from the review question?** | CONCERN: LOW/HIGH/UNCLEAR |
| **Domain 3: Reference standard** | |
| 1. **Risk of bias** |  |
| **Describe the reference standard and how it was conducted and interpreted:** | |
| - **Is the reference standard likely to correctly classify the target condition?** | Yes/No/Unclear |
| - **Were the reference standard results interpreted without knowledge of the results of the index test?** | Yes/No/Unclear |
| **Could the reference standard, its conduct, or its interpretation have introduced bias?** | RISK: LOW/HIGH/UNCLEAR |
| 1. **Concerns regarding applicability** |  |
| **Is there concern that the target condition as defined by the reference standard does not match the review question?** | CONCERN: LOW/HIGH/UNCLEAR |
| **Domain 4: Flow and timing** | |
| 1. **Risk of bias** |  |
| **Describe any patients who did not receive the index test(s) and/or reference standard or who were excluded from the 2x2 table (refer to flow diagram):**  16 patients were excluded (technical or protocol issues). The rest of the patients received both index and reference tests.  **Describe the time interval and any interventions between index test(s) and reference standard:**  Both scans performed during the same clinical encounter. | |
| - **Was there an appropriate interval between index test(s) and reference standard?** | Yes/No/Unclear |
| - **Did all patients receive a reference standard?** | Yes/No/Unclear/ |
| - **Did patients receive the same reference standard?** | Yes/No/Unclear |
| - **Were all patients included in the analysis?** | Yes/No/Unclear |
| **Could the patient flow have introduced bias?** | RISK: LOW/HIGH/UNCLEAR |

Curry et al 2025

QUADAS-2 tool: Risk of bias and applicability judgment

| **Domain 1: Patient selection** | |
| --- | --- |
| 1. **Risk of bias** |  |
| **Describe methods of patient selection:**  Consecutive patients presenting to the Emergency Department (ED) across 11 United Kingdom (UK) hospitals with clinical suspicion of deep vein thrombosis (DVT) were enrolled prospectively. | |
| - **Was a consecutive or random sample of patients enrolled?** | Yes/No/Unclear |
| - **Was a case-control design avoided?** | Yes/No/Unclear |
| - **Did the study avoid inappropriate exclusions?** | Yes/No/Unclear |
| **Could the selection of patients have introduced bias?** | RISK: LOW/HIGH/UNCLEAR |
| 1. **Concerns regarding applicability** |  |
| **Describe included patients (prior testing, presentation, intended use of index test and setting):**  Patients with suspectived DVT presenting to the ED across 11 UK hospitals were recruited prospectively. Index test was intended as a point-of-care test at the ED. The handheld ultrasound device using built-in artificial intelligence (AI) at the common femoral and popliteal veins.  No prior imaging before the AI scan. | |
| **Is there concern that the included patients do not match the review question?** | CONCERN: LOW/HIGH/UNCLEAR |
| **Domain 2: Index test(s) *(if more than 1 index test was used, please complete for each test)*** | |
| 1. **Risk of bias** |  |
| **Describe the index test and how it was conducted and interpreted:**  The index test was AutoDVT, an AI-guided two-point compression ultrasound system designed to detect proximal deep vein thrombosis (DVT) when used by non-experats. The non-experts received a 60-minute training session and used a handheld ultrasound device paired with AutoDVT software to scan the common femoral and popliteal veins. | |
| - **Were the index test results interpreted without knowledge of the results of the reference standard?** | Yes/No/Unclear |
| - **If a threshold was used, was it pre-specified?** | Yes/No/Unclear  (ACEP > =3 = sufficient quality) |
| **Could the conduct or interpretation of the index test have introduced bias?** | RISK: LOW/HIGH/UNCLEAR |
| 1. **Concerns regarding applicability** |  |
| **Is there concern that the index test, its conduct, or interpretation differ from the review question?** | CONCERN: LOW/HIGH/UNCLEAR |
| **Domain 3: Reference standard** | |
| 1. **Risk of bias** |  |
| **Describe the reference standard and how it was conducted and interpreted:**  In the study by Curry et al. (2025), the reference standard was a formal compression ultrasound (USS) performed by trained radiographers or radiologists as part of routine clinical care. | |
| - **Is the reference standard likely to correctly classify the target condition?** | Yes/No/Unclear |
| - **Were the reference standard results interpreted without knowledge of the results of the index test?** | Yes/No/Unclear |
| **Could the reference standard, its conduct, or its interpretation have introduced bias?** | RISK: LOW/HIGH/UNCLEAR |
| 1. **Concerns regarding applicability** |  |
| **Is there concern that the target condition as defined by the reference standard does not match the review question?** | CONCERN: LOW/HIGH/UNCLEAR |
| **Domain 4: Flow and timing** | |
| 1. **Risk of bias** |  |
| **Describe any patients who did not receive the index test(s) and/or reference standard or who were excluded from the 2x2 table (refer to flow diagram):**  16 patients were excluded (technical or protocol issues). The rest of the patients received both index and reference tests.  **Describe the time interval and any interventions between index test(s) and reference standard:**  Both scans performed during the same clinical encounter. | |
| - **Was there an appropriate interval between index test(s) and reference standard?** | Yes/No/Unclear |
| - **Did all patients receive a reference standard?** | Yes/No/Unclear/ |
| - **Did patients receive the same reference standard?** | Yes/No/Unclear |
| - **Were all patients included in the analysis?** | Yes/No/Unclear |
| **Could the patient flow have introduced bias?** | RISK: LOW/HIGH/UNCLEAR |

Speranza et al 2025

QUADAS-2 tool: Risk of bias and applicability judgment

| **Domain 1: Patient selection** | |
| --- | --- |
| 1. **Risk of bias** |  |
| **Describe methods of patient selection:**  381 patients were enrolled for the study. 294 patients with proximal deep vein thrombosis (DVT), 15 patients with distal DVT, 10 patients with protocol deviations, 62 patients who were recruited prior to minor adjustments. | |
| - **Was a consecutive or random sample of patients enrolled?** | Yes/No/Unclear |
| - **Was a case-control design avoided?** | Yes/No/Unclear |
| - **Did the study avoid inappropriate exclusions?** | Yes/No/Unclear |
| **Could the selection of patients have introduced bias?** | RISK: LOW/HIGH/UNCLEAR |
| 1. **Concerns regarding applicability** |  |
| **Describe included patients (prior testing, presentation, intended use of index test and setting):**  Consecutive patients with suspected DVT were recruited prospectively at 11 United Kingdom (UK) hospitals. The patients were excluded if > 12 weeks pregnant or had ipsilateral deep vein thrombosis (DVT). No formal ultrasound performed prior. The study used ThinkSono Guidance softward with Clarius L HD probe. The intention was to triage with AI-guided ultrasound by non-experts after one hour of training. The results were reviewed by 5 radiologists and 5 Emergency Medicine doctors. | |
| **Is there concern that the included patients do not match the review question?** | CONCERN: LOW/HIGH/UNCLEAR |
| **Domain 2: Index test(s) *(if more than 1 index test was used, please complete for each test)*** | |
| 1. **Risk of bias** |  |
| **Describe the index test and how it was conducted and interpreted:**  AI-guided scan was performed by nurses using standardized protocol. Remote review was then performed by blinded radiologists or EM physicians. Diagnostic quality was determined by ACEP score. | |
| - **Were the index test results interpreted without knowledge of the results of the reference standard?** | Yes/No/Unclear |
| - **If a threshold was used, was it pre-specified?** | Yes/No/Unclear  (ACEP > =3 = sufficient quality) |
| **Could the conduct or interpretation of the index test have introduced bias?** | RISK: LOW/HIGH/UNCLEAR |
| 1. **Concerns regarding applicability** |  |
| **Is there concern that the index test, its conduct, or interpretation differ from the review question?** | CONCERN: LOW/HIGH/UNCLEAR |
| **Domain 3: Reference standard** | |
| 1. **Risk of bias** |  |
| **Describe the reference standard and how it was conducted and interpreted:**  Formal compression ultrasound was performed by trained sonographers at each site, consistent with routine clinical practice. | |
| - **Is the reference standard likely to correctly classify the target condition?** | Yes/No/Unclear |
| - **Were the reference standard results interpreted without knowledge of the results of the index test?** | Yes/No/Unclear |
| **Could the reference standard, its conduct, or its interpretation have introduced bias?** | RISK: LOW/HIGH/UNCLEAR |
| 1. **Concerns regarding applicability** |  |
| **Is there concern that the target condition as defined by the reference standard does not match the review question?** | CONCERN: LOW/HIGH/UNCLEAR |
| **Domain 4: Flow and timing** | |
| 1. **Risk of bias** |  |
| **Describe any patients who did not receive the index test(s) and/or reference standard or who were excluded from the 2x2 table (refer to flow diagram):**  All patients received both scans. However, only 80% of scans met diagnostic quality for expert review.  **Describe the time interval and any interventions between index test(s) and reference standard:**  Both scans were performed during the same clinical encounter. | |
| - **Was there an appropriate interval between index test(s) and reference standard?** | Yes/No/Unclear |
| - **Did all patients receive a reference standard?** | Yes/No/Unclear/ |
| - **Did patients receive the same reference standard?** | Yes/No/Unclear |
| - **Were all patients included in the analysis?** | Yes/No/Unclear |
| **Could the patient flow have introduced bias?** | RISK: LOW/HIGH/UNCLEAR |

Avgerinos et al 2025

QUADAS-2 tool: Risk of bias and applicability judgment

| **Domain 1: Patient selection** | |
| --- | --- |
| 1. **Risk of bias** |  |
| **Describe methods of patient selection:**  Prospective enrollment of consecutive patients with suspected DVT at the emergency department of Attiikon University Hospital in Athens, Greece over 12 months. | |
| - **Was a consecutive or random sample of patients enrolled?** | Yes/No/Unclear |
| - **Was a case-control design avoided?** | Yes/No/Unclear |
| - **Did the study avoid inappropriate exclusions?** | Yes/No/Unclear |
| **Could the selection of patients have introduced bias?** | RISK: LOW/HIGH/UNCLEAR |
| 1. **Concerns regarding applicability** |  |
| **Describe included patients (prior testing, presentation, intended use of index test and setting):**  Consecutive patients with suspected DVT were recruited prospectively at the emergency department of Attikon University at Athens, Greece. Recruitment was done over 12 months. ThinkSono Gidance System was used. Operators were non-vascular trained ED doctors. Intended use of the system is for the detection of proximal limb DVT. | |
| **Is there concern that the included patients do not match the review question?** | CONCERN: LOW/HIGH/UNCLEAR |
| **Domain 2: Index test(s) *(if more than 1 index test was used, please complete for each test)*** | |
| 1. **Risk of bias** |  |
| **Describe the index test and how it was conducted and interpreted:**  Formal duplex ultrasound performed per clinical standard by experienced vascular sonographers or radiologists, with interpretation as part of routine workflow. | |
| - **Were the index test results interpreted without knowledge of the results of the reference standard?** | Yes/No/Unclear |
| - **If a threshold was used, was it pre-specified?** | Yes/No/Unclear |
| **Could the conduct or interpretation of the index test have introduced bias?** | RISK: LOW/HIGH/UNCLEAR |
| 1. **Concerns regarding applicability** |  |
| **Is there concern that the index test, its conduct, or interpretation differ from the review question?** | CONCERN: LOW/HIGH/UNCLEAR |
| **Domain 3: Reference standard** | |
| 1. **Risk of bias** |  |
| **Describe the reference standard and how it was conducted and interpreted:**  AI-guided handheld ultrasound performed by non-vascular trained ED clinicians using a standardized two-point compression protocol at the groin (common femoral vein, femoral and profunda femoral vein) and popliteal fossa (popliteal vein, proximal trifurcatio of the distal veins). Images were reviewed independently by two blinded expert reviewers. | |
| - **Is the reference standard likely to correctly classify the target condition?** | Yes/No/Unclear |
| - **Were the reference standard results interpreted without knowledge of the results of the index test?** | Yes/No/Unclear |
| **Could the reference standard, its conduct, or its interpretation have introduced bias?** | RISK: LOW/HIGH/UNCLEAR |
| 1. **Concerns regarding applicability** |  |
| **Is there concern that the target condition as defined by the reference standard does not match the review question?** | CONCERN: LOW/HIGH/UNCLEAR |
| **Domain 4: Flow and timing** | |
| 1. **Risk of bias** |  |
| **Describe any patients who did not receive the index test(s) and/or reference standard or who were excluded from the 2x2 table (refer to flow diagram):**  7 patients were excluded from withdrawn or incomplete studies.  **Describe the time interval and any interventions between index test(s) and reference standard:**  Both scans were performed during the same clinical encounter. | |
| - **Was there an appropriate interval between index test(s) and reference standard?** | Yes/No/Unclear |
| - **Did all patients receive a reference standard?** | Yes/No/Unclear/ |
| - **Did patients receive the same reference standard?** | Yes/No/Unclear |
| - **Were all patients included in the analysis?** | Yes/No/Unclear |
| **Could the patient flow have introduced bias?** | RISK: LOW/HIGH/UNCLEAR |
