# Supplementary material for: Artificial intelligence in the diagnosis of deep vein thrombosis: A scoping review
Source: PLoS One. 2026 Jun 22;21(6):e0351558. doi: 10.1371/journal.pone.0351558 (PMC13286142; doi:10.1371/journal.pone.0351558)
Supplement: S4 Appendix — (DOCX) [file pone.0351558.s004.docx]

**Fundamentals of artificial intelligence (AI)**

**Deep learning**

Deep learning, a subset of artificial intelligence (AI) and machine learning (ML), uses layers of neurons connected by “weights.” Each neuron functions as a single linear regression unit, generating outputs from inputs passed forward during training [2, 19]. The disparity between the network’s final output and the true label enables the estimation of error. This error estimation guides the algorithm in adjusting the network’s weights and optimizing its performance through a process known as backpropagation [2, 19]. Additionally, backpropagation strengthens accurate network connections while attenuating irrelevant ones. The algorithm reduces the discrepancy between the output and true label, thereby lowering the overall network error rate [2, 19].

**Convolutional neural network (CNNs)**

Convolutional neural networks (CNN) are a widely used deep learning model for imaging analyses. Each CNN layer contains filters, which are small matrices of weights that are applied to image pixels [2, 19]. These filters are responsible for pattern recognition across images, utilizing both shallow and deeper layers of the network. The shallow layers of the CNN examine low-level geometric patterns such as lines and circles in the images [2, 19]. The deeper layers of the CNN have a high level of understanding for the context of the image (i.e., “image with DVT” vs. “image without DVT”) [2, 19, 20].

RetinaNet is a common convolutional neural network designed to detect multiple small objects efficiently and accurately in images via a single pass through the network. Its applications are commonly observed in the detection of tumors and small lesions in medical images [21, 22].
