# Supplementary material for: Artificial intelligence in the diagnosis of deep vein thrombosis: A scoping review
Source: PLoS One. 2026 Jun 22;21(6):e0351558. doi: 10.1371/journal.pone.0351558 (PMC13286142; doi:10.1371/journal.pone.0351558)
Supplement: S1 Table — (DOCX) [file pone.0351558.s005.docx]

**Supplement 4. Sensitivity, specificity, accuracy, and precision of AI models on CT, US, and MRI**

| **Literature** | **Imaging Modality** | **AI Technique** | **Sensitivity** | **Specificity** | **Accuracy** | | **PPV** | **NPV** | **Precision** |
| --- | --- | --- | --- | --- | --- | --- | --- | --- | --- |
| Kainz et al. 2021 | Ultrasound | Deep learning Model for compression US Videos | 0.82-0.96 | 0.70 -0.82 | 0.75-0.83 | 0.65-0.89 | | 0.98-0.99 | -- |
| Sun et al. 2021 | Black-Blood magnetic resonance (MR) | Deep learning network for Venous thrombus segmentation | Internal testing:  0.95 +/- 0.07  External testing:  0.93 +/- 0.06 on 3.0T DANTE SPACE  0.81 +/- 0.12 on 3.0T DANTE FLASH | Internal testing:  0.97 +/- 0.02  External Testing:  0.92 +/- 0.09 on 3.0T DANTE SPACE  097 +/- 0 0.02 on 3.0T DANTE FLASH | Internal Testing:  0.96 +/- 0.03  External Testing:  0.94 +/- 0.05 on 3.0T DANTE SPACE  0.95 +/- 0.04 on 3.0T DANTE FLASH | Internal Testing:  0.97 +/- 0.04  External Testing:  0.82 +/- 0.18 on 3.0 T DANTE SPACE  0.96 +/- 0.10 on 3.0T DANTE FLASH | | Internal Testing:  0.97 +/-0.04  External Testing:  0.96 +/-0.02 on 3.0T DANTE SPACE  0.94 +/- 0.05 on 3.0T DANTE FLASH | -- |
| Nakayama et al. 2022 | Ultrasound (US) | Deep learning to automatically identify US images of popliteal vein | -- | -- | 0.76 for portable US 0.72 for stationary US | -- | | -- | -- |
| Seo et al. 2023 | Computed Tomography (CT) Angiography | Convolutional neural network based RetinaNet Model to detect DVT on synthesized images | 0.833 (0.826-0839) for ResNet152  0.831(0.819-0.843) for ResNet50 | -- | -- | -- | | -- | 0.660 (0.650-0.670) for ResNet152  0.632 (0.610-0.654) for ResNet50 |
| Oppenheimer et al. 2023 | Ultrasound  (US) | Machine-learning software to assist in the diagnosis of DVT | 1.00 | 0.95 | -- | -- | | -- | -- |
| Joseph et al. 2024 | Computed Tomography (CT) & Magnetic Resonance Imaging (MRI) | CNN + Sooty tern Optimization diagnose DVT | -- | 0.9608 | 0.9608 | -- | | -- | 0.9714 |
| Nothnagel & Aslam 2024 | Ultrasound  (US) | ThinkSono to assist in the diagnosis of DVT | 0.99-1.00 (1.00) | 0.90-0.92 (0.90) | -- | -- | | -- | -- |
| Arun et al 2025 | Ultrasound (US), Computed Tomography (CT), and Magnetic Resonance Imaging (MRI) | Deep R-Belief Neural Network to diagnose DVT | -- | 0.967 | 0.989 | -- | | -- | 0.975 |
| Curry et al. 2025 | Ultrasound  (US) | AutoDVT to assist in the diagnosis of DVT | 0.49-0.83 (0.68) | 0.74-0.85 (0.8) | -- | 0.19-0.40 (0.28) | | 0.92-0.98 (95) | -- |
| Speranza et al. 2025 | Ultrasound (US) | ThinkSono to assist in diagnosis of DVT | Radiologist:  0.9-0.95  EM Reviewers:  0.95-0.98 | Radiologist:  0.74-0.84  EM Reviewers:  0.97-1.00 | -- | Radiologist:  0.30-0.42  EM Reviewers:  0.81-1.00 | | Radiologist:  0.98-0.99  EM Reviewers:  0.99 | -- |
| Avgerinos et al 2025 | Ultrasound  (US) | ThinkSono to assist in diagnosis of DVT | 1.00 | 0.95 | -- | 0.75 | | 1.00 | -- |
